# Menopause and Mental Health: Improving Knowledge and Clinical Practice in a Community Mental Health Team–A Quality Improvement Project

**DOI:** 10.1192/bjo.2026.11442

**Published:** 2026-06-30

**Authors:** Mitra Nasri, Stephanie Allan, Resha Jazrawi

**Affiliations:** North London NHS Foundation Trust, London, United Kingdom

## Abstract

**Aims:**

A HSIB (2023) report highlights the substantial impact of menopause on mental health, with up to 40% experiencing depressive symptoms and a seven-fold increase in suicidal ideation risk. Despite this, menopause remains under-recognised in community mental health team (CMHT) psychiatric assessments, with symptoms often misattributed to, or exacerbating, severe mental illness. This quality improvement project aimed to enhance CMHT clinicians’ awareness and knowledge of the menopause–mental health link, targeting a 50% increase within the Barnet North Core CMHT, measured through clinicians’ understanding of its relevance to mental health, incorporation into assessments, and knowledge of NICE treatments guidelines.

**Methods:**

Baseline knowledge of menopause and mental health among 35 CMHT healthcare professionals (HCPs) was assessed using a questionnaire. Targeted educational training was delivered to address identified knowledge gaps and support improved integration of menopause considerations into mental health assessments. Outcomes were measured via pre-training and post-training questionnaires. Supplementary interventions included: (1) review of existing Trust resources, (2) promotion of patient leaflets, (3) provision of screening and diagnostic tools, (4) development of a narrated menopause training module, and (5) display of reminder posters to reinforce learning.

**Results:**

All HCPs recognised the relevance of menopause to mental health pre- and post-training (100%), with awareness of its impact increasing slightly from 74% to 80%. Self-rated sufficient knowledge increased from 16% to 40%, although most continued to desire further training (100% pre; 93% post). Consideration of menopause in assessments was largely unchanged (68% to 60%), while incorporation into mental state examinations improved markedly (42% to 87%). Knowledge of appropriate treatment increased substantially from 23% to 80%, and awareness of available resources also improved, though gaps remained (8% to 40%).

**Conclusion:**

Targeted CMHT training improved awareness of menopause’s impact on mental health, alongside enhanced knowledge of associated risks, clinical integration into mental state examinations and knowledge of NICE treatment guidelines. Despite these gains, only 40% of HCPs reported sufficient knowledge post-intervention, and 93% expressed a desire for further training.

These findings underscore the need for sustained educational interventions, particularly in the context of staff turnover, which may have limited engagement. To support sustainability, a narrated training module, validated tools, patient leaflets, and reminder posters were implemented. Persistent knowledge gaps highlight the importance of ongoing training, embedding menopause–mental health content within mandatory Trust programmes, and evaluating the impact of sustainability measures on improving knowledge and application of the menopause–mental health link in clinical practice.

